# Senescence in Post-Mitotic Cells: A Driver of Aging?

**DOI:** 10.1089/ars.2020.8048

**Published:** 2021-01-08

**Authors:** Thomas von Zglinicki, Tengfei Wan, Satomi Miwa

**Affiliations:** ^1^ Ageing Research Laboratories, Faculty of Medical Sciences, Biosciences Institute, Newcastle University, Newcastle upon Tyne, United Kingdom.; ^2^ Molecular Biology and Genetics, Arts and Sciences Faculty, Near East University, Nicosia, Turkey.

**Keywords:** aging, senescence, post-mitotic

## Abstract

***Significance:*** Cell senescence was originally defined by an acute loss of replicative capacity and thus believed to be restricted to proliferation-competent cells. More recently, senescence has been recognized as a cellular stress and damage response encompassing multiple pathways or senescence domains, namely DNA damage response, cell cycle arrest, senescence-associated secretory phenotype, senescence-associated mitochondrial dysfunction, autophagy/mitophagy dysfunction, nutrient and stress signaling, and epigenetic reprogramming. Each of these domains is activated during senescence, and all appear to interact with each other. Cell senescence has been identified as an important driver of mammalian aging.

***Recent Advances:*** Activation of all these senescence domains has now also been observed in a wide range of post-mitotic cells, suggesting that senescence as a stress response can occur in nondividing cells temporally uncoupled from cell cycle arrest. Here, we review recent evidence for post-mitotic cell senescence and speculate about its possible relevance for mammalian aging.

***Critical Issues:*** Although a majority of senescence domains has been found to be activated in a range of post-mitotic cells during aging, independent confirmation of these results is still lacking for most of them.

***Future Directions:*** To define whether post-mitotic senescence plays a significant role as a driver of aging phenotypes in tissues such as brain, muscle, heart, and others. *Antioxid. Redox Signal.* 34, 308–323.

## What Is Senescence?

Cellular senescence was first discovered and defined by Hayflick and Moorhead in 1961 to describe the phenomenon of limited proliferation capacity in cultured human fibroblasts (50). This replicative senescence (RS) was found to be mainly induced by the dysfunction and critical shortening of telomeres (9, 27, 136). With more studies being conducted on senescence, the understanding and the definition have been expanded and evolved. For example, stressors, such as DNA damage, oxidative stress, and oncogene activation, were also found to trigger permanent cell cycle arrest and this phenomenon was named as stress-induced premature senescence (SIPS) (16, 82, 119, 129). It was often assumed that RS would be dependent on telomere shortening, whereas SIPS would be telomere length independent. However, this distinction is much less straightforward than originally believed, because stresses can either accelerate telomere shortening (130, 131), thus inducing RS prematurely (84, 107), or uncap telomeres without any shortening (51), causing telomere-dependent SIPS. This led to the suggestion that all forms of senescence are cellular stress responses (132), which is now generally accepted (48). Although senescence plays important beneficial roles for tissue remodeling during embryonic development (96, 125), in tissue repair and wound healing (33, 75) and as a tumor suppressor (117), it has become abundantly clear that the accumulation of senescent cells in multiple, if not all, tissues is a major driver of degenerative phenotypes, disabilities, and diseases during aging (5, 121, 140).

**FIG. 1.**
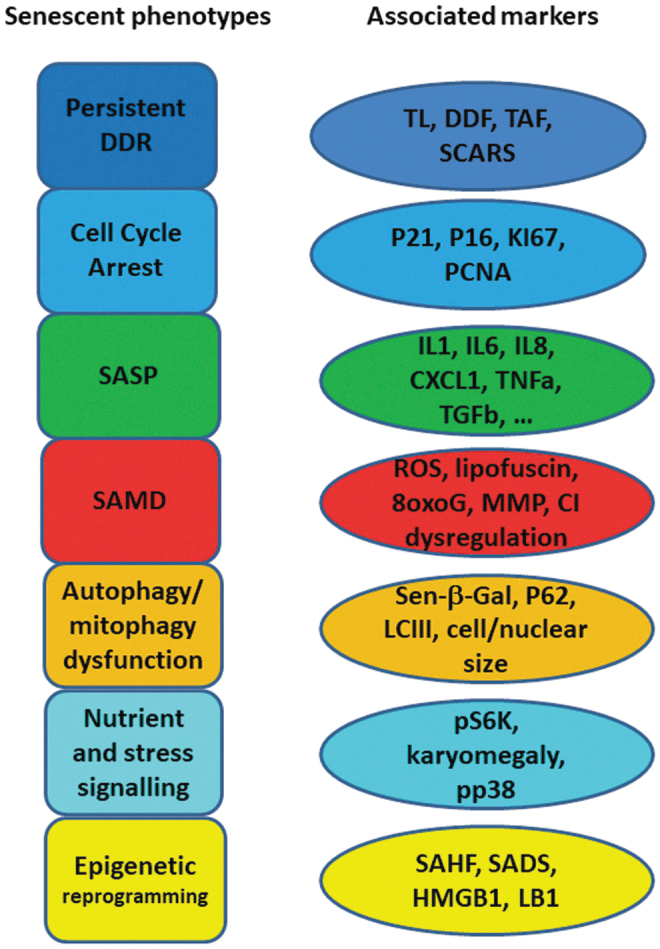
**Phenotypes (“building blocks”) of the senescent state and observable markers associated with it.** Although all phenotypes are tightly interrelated (not shown), individual phenotypes might be more or less strongly activated in individual senescent cells depending on contexts including senescence inducer, cell type, tissue environment, and others. For each phenotype, there are multiple markers that enable assessment of its involvement in a given senescent state. CI, complex I of the mitochondrial electron transport chain; DDF, DNA damage foci; DDR, DNA damage response; ROS, reactive oxygen species; SADS, senescence-associated distension of satellites; SAHF, senescence-associated heterochromatin foci; SAMD, senescence-associated mitochondrial dysfunction; SASP, senescence-associated secretory phenotype; SCARS, DNA segments with chromatin alterations reinforcing senescence; TAF, telomere-associated foci; TL, telomere length. Color images are available online.

Recent studies have shown that senescence is much more than an extended cell cycle arrest (2, 48, 66, 106). Several “building blocks” or phenotypes participate and interplay in senescence, forming positive feedback loops that trigger, develop, and maintain the senescent cell state (Fig. 1).

A persistent DNA damage response (DDR) is probably the most consistently observed feature of senescent cells. Although it can be triggered and maintained by non-telomeric DNA damage (106), the repair deficiency of telomeres (36, 108, 112) makes them prime candidates for inducers of the senescent DDR (51). The main DDR downstream pathway comprises Ataxia telangiectasia mutated (ATM) and ATM and RAD3-related (ATR) kinases, which belong to the phosphatidylinositol 3-kinase like family of protein kinases (71, 80). Activated ATM/ATR subsequently activate Chk1 and Chk2, leading to p53 phosphorylation and stabilization and transcriptional induction of the cyclin-dependent kinase (CDK) inhibitor p21, which then inhibits CDK1/2 and Cyclin A/E complexes (17, 21). Their inhibition triggers the hypo-phosphorylation of Rb, eventually leading to cell cycle arrest (21). In addition, upregulation of gene expression from the CDKN2A locus, mainly p16^INK4a^ and p14^ARF^ (human)/p19^ARF^ (mouse), is also an initiator of senescence (45, 63, 65). Repression of these genes in young cells is released in tissue during aging due to the loss of the polycomb repressive complexes (11, 55) *via* unclear mechanisms. p14/p19^ARF^ can inactivate the p53-degrading E3 ubiquitin protein ligase MDM2 and, therefore, maintain p53 at a high level (45, 63); whereas p16^INK4a^, a CDK inhibitor, can directly inhibit CDK4, 6 and cyclin D complexes, leading to hypo-phosphorylation of Rb and cell cycle arrest (45, 63).

Although these pathways initiate the cell cycle arrest, they are usually not sufficient to keep it persistent, which in most cells requires the induction both of the senescence-associated secretory phenotype (SASP) (2, 66) and of senescence-associated mitochondrial dysfunction (SAMD) (106). The SASP comprises a variety of secreted factors. These include preferentially proinflammatory cytokines and chemokines, growth factors, and matrix-modifying factors that are typically driven by nuclear factor-κB (NF-κB) and CCAAT/enhancer binding Protein-β transcription factors. A second arm of the SASP is preferentially immunosuppressive and fibrogenic and dependent on Wnt/Notch-1, mainly producing transforming growth factor beta (TGFβ) (52); whereas a third damage-associated molecular patterns-dependent arm secretes acetylated HMGB1, heat shock proteins, oxidized lipids, reactive oxygen species (ROS) (*e.g.*, hydrogen peroxide), DNA, and microRNAs, which are often associated with exosomes (10, 22, 23, 57, 79, 127). The production and secretion of SASP facilitates the maintenance and spreading of senescence by autocrine and paracrine mechanisms. This, on one hand, promotes tissue remodeling and repairing (33, 75, 95, 96, 125), but on the other hand, it may contribute to tissue functional decline, age-related tissue degeneration, and tumorigenesis (22, 31, 56, 67, 87).

The DDR can initiate SAMD *via* p38MAPK and TGFβ pathways (8, 64, 94, 106) or by SASP *via* upregulation of NF-κB (61). SAMD is characterized by the concomitant increase in mitochondrial uncoupling (and decrease in mitochondrial membrane potential) together with hyperproduction of ROS (106, 107). Enhanced ROS, in turn, facilitate more DNA damage (106) and SASP secretion (24), thus driving positive, senescence-stabilizing feedback loops. Moreover, SAMD may also promote mechanistic/mammalian target of rapamycin (mTOR) activity independent of nutrition deprivation in senescent cells (99), which contributes to a constant high level of mTOR activity, the nutrient signaling feature of senescence (19, 139).

The contribution of mTOR activity to senescence has been hypothesized *via* the competitive binding of mTOR complexes (mTORC) 1 and 2 to p53, leading to p53 phosphorylation at serine 15 and activation of downstream pathways for the development of senescence (59). A high level of mTOR, especially mTORC1 activity, also suppresses autophagy, which has been suggested in some studies to induce senescence (62, 81, 126). However, the role of autophagy in senescence is still controversial, as senescence can also be promoted by overexpression of the autophagy-related gene ULK3 and suppressed by knockdown of autophagy core genes Atg5 and 7 (101, 138). Importantly, mitochondria-specific autophagy (mitophagy) is consistently downregulated in senescence, probably due to mTORC1 over-activation (64). This disturbs the balance between mitochondrial biogenesis and turnover, resulting in the accumulation of dysfunctional, damaged mitochondria, leading to more ROS generation and enhanced DDR (30). So far, it is not yet clear whether mitophagy decline is the primary driver for SAMD, or whether SAMD start before declining mitophagy during senescence induction. The accumulation of lipofuscin (non-degradable, highly autofluorescent residual lysosomal content, consisting of cross-linked oxidized lipids and proteins) due to lysosomal overload is also a feature of senescence (123). Lysosomal accumulation of lipofuscin may impair not only autophagy and mitophagy but also the proteasome (122), forming another senescence-stabilizing feedback between multiple features of senescence.

Finally, epigenetic reprogramming and chromatin reorganization occur during senescence, driven, for instance, by mitochondrial retrograde response (15, 107) and increased lamin B1 (LB1) degradation by abnormal autophagy (37, 40). Markers of epigenetic reprogramming in senescence are: the appearance of senescence-associated heterochromatin foci, domains of facultative heterochromatin that contribute to silencing of proliferation-promoting genes (100), and changes (generally lower levels) in DNA and histone methylation, specifically H3K9me3 and H3K27me3 (115). The epigenetic reprogramming stabilizes other senescence building blocks by promoting mitochondrial dysfunction (47) and SASP production (18, 120).

In conclusion, and in agreement with a recent consensus (48), we define cell senescence as a cellular response to a wide variety of stresses (including replicative, oncogenic, oxidative, DNA damage, developmental and other stresses) in which the phenotypes indicated earlier generate a senescence-stabilizing interaction network. Individual phenotypes might be weakly expressed or even absent, as long as the majority of them ensures a sufficiently stable network to maintain the cell senescent state.

Such an understanding of the cell senescence program allows for the temporal separation between a full senescence phenotype and cell cycle arrest. We believe that this may be important in two different ways. First, tumor cells can be induced to senesce by DNA-damaging therapies; however, after having developed a senescent phenotype, some of these revert from cell cycle arrest and resume cycling, but they appear to retain features of SASP, SAMD, and senescent epigenetic reprogramming. Importantly, these features render these previously senescent cells very similar to cancer stem cells, providing increased proliferative and metastatic potential (26, 90, 91, 133). Thus, although it may simply be a semantic distinction whether to call these reverted cells senescent, it would be of major practical importance to understand how some features of senescence can be retained during resumption of proliferation.

Second, this understanding of the senescence program enables us to use the huge accumulated body of knowledge about cellular senescence to better understand stress responses and cell aging mechanisms in post-mitotic cells. Post-mitotic cells are essential for the function of major tissues, including brain, heart, and skeletal muscle. There are preliminary observations of a senescent phenotype developing and increasing during aging in these tissues, which we will review later. Importantly, how different mechanisms that promote aging, including DNA damage, chronic inflammation, and mitochondrial dysfunction, are interlinked in these tissues is still not well understood. Obviously, if post-mitotic senescence is relevant, translation of knowledge from the cell senescence field might lead to very interesting and novel clues regarding on how such tissues are aging. We will speculatively address this possibility in the final part of this review.

## Senescence in Post-Mitotic Cells: The Present Evidence

To our knowledge, our group published the first study describing a “senescence-like phenotype” in post-mitotic cells (60). This work showed that multiple senescence markers were found together in the same neurons (in both the central nervous system and peripheral ones) from old, but not from young, mice. Importantly, by using single- and double-gene knock-out mice, we also showed that accumulation of neurons bearing senescence markers was initiated by a DDR driven by dysfunctional telomeres and was dependent on signaling through p21 (60).

**FIG. 2.**
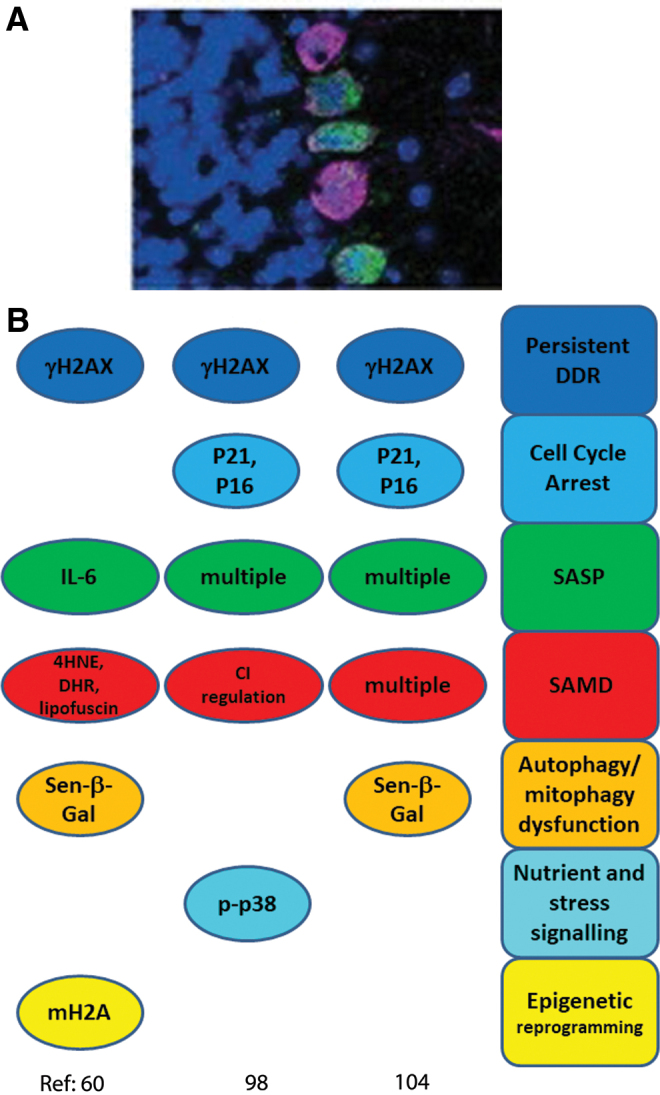
**Senescence markers measured in neurons. (A)** Purkinje neurons (labeled by calbindin, *purple*) in an old (32 months) mouse frequently stain positive for IL-6 (*green*) (60). **(B)** Senescence markers observed and senescence phenotypes inferred in neurons (60, 98, 104). IL, interleukin. Color images are available online.

Since this seminal study, accumulation of multiple senescence markers in aging mice has been shown for major post-mitotic cells types residing in different tissues, such as retinal ganglion cells, cardiomyocytes, skeletal myofibers, cochlear cells, and osteocytes (Fig. 2). Most of these results are very recent, and were not covered by other reviews (114). So far, only in the case of neurons, confirmatory data have been presented by independent laboratories (60, 98, 104); whereas our knowledge about post-mitotic senescence in other tissues is still only based on a single publication per tissue. Moreover, information about some important cell types is completely lacking. For instance, there is no doubt about occurrence and functional relevance of cell senescence in fat tissue (20, 54, 128, 135). However, it remains unclear whether this is restricted to preadipocytes or whether terminally differentiated adipocytes are able/prone to senesce as well. Moreover, to our knowledge, there are no data yet on the role of cell senescence in aging of organisms made up preferentially of post-mitotic cells such as *Caenorhabditis elegans* or *Drosophila melanogaster*.

**FIG. 3.**
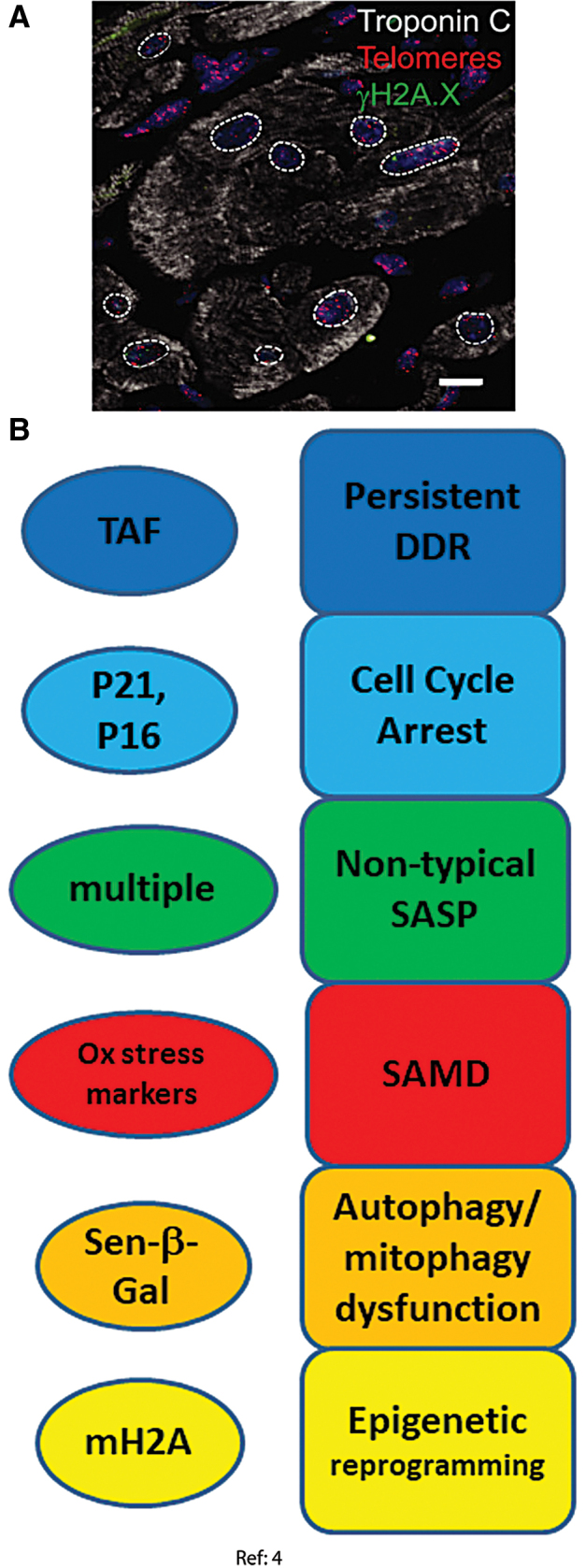
**Senescence markers measured in cardiomyocytes. (A)** Mouse cardiomyocytes (labelled with Troponin C, *white*) show co-localization of telomeres (*red*) and γH2AX-positive DDF (*green*) (4). Cardiomyocyte nuclei are labelled with *dotted lines*. **(B)** Senescence markers observed and senescence phenotypes inferred in cardiomyocytes (4). Color images are available online.

**FIG. 4.**
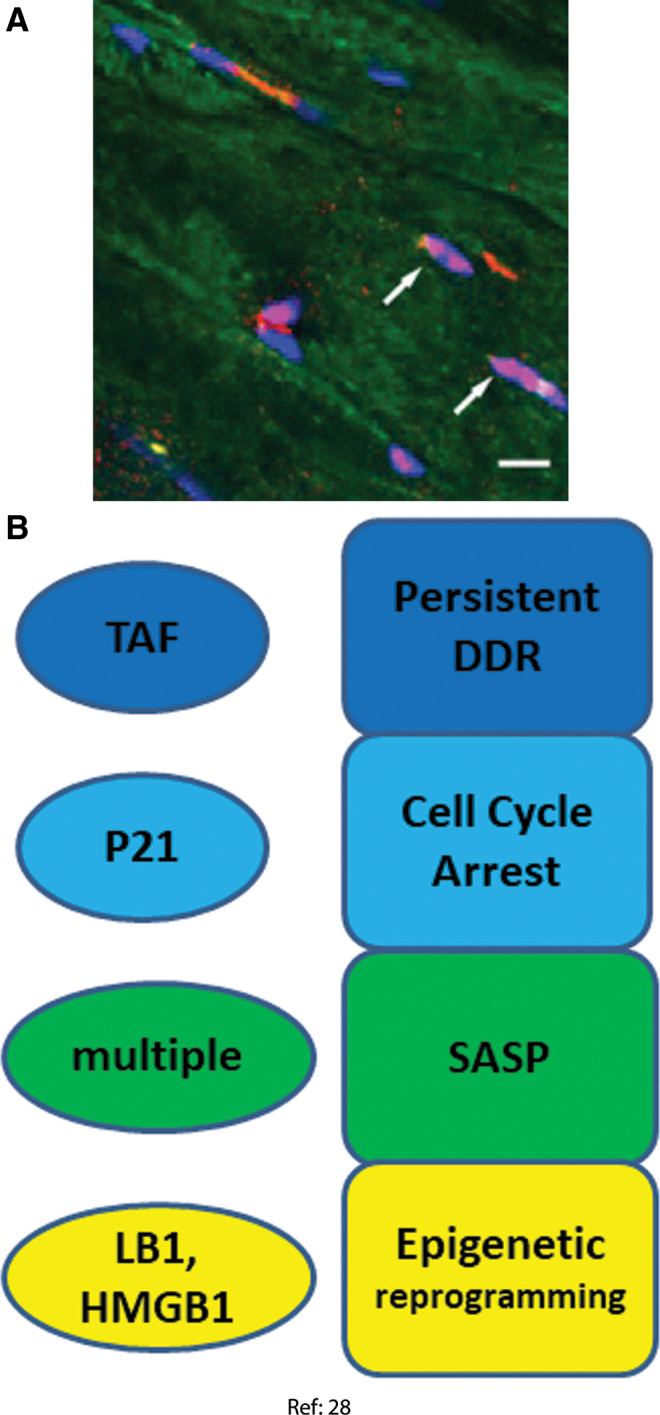
**Senescence markers measured in skeletal muscle myofibers. (A)** Gastrocnemius muscle from a 32-month-old mouse. *Blue*: DAPI, *red*: p21, *green*: autofluorescence (28). *White arrows* indicate p21-positive centrally located nuclei. **(B)** Senescence markers observed and senescence phenotypes inferred in skeletal myofibers (28). Color images are available online.

**FIG. 5.**
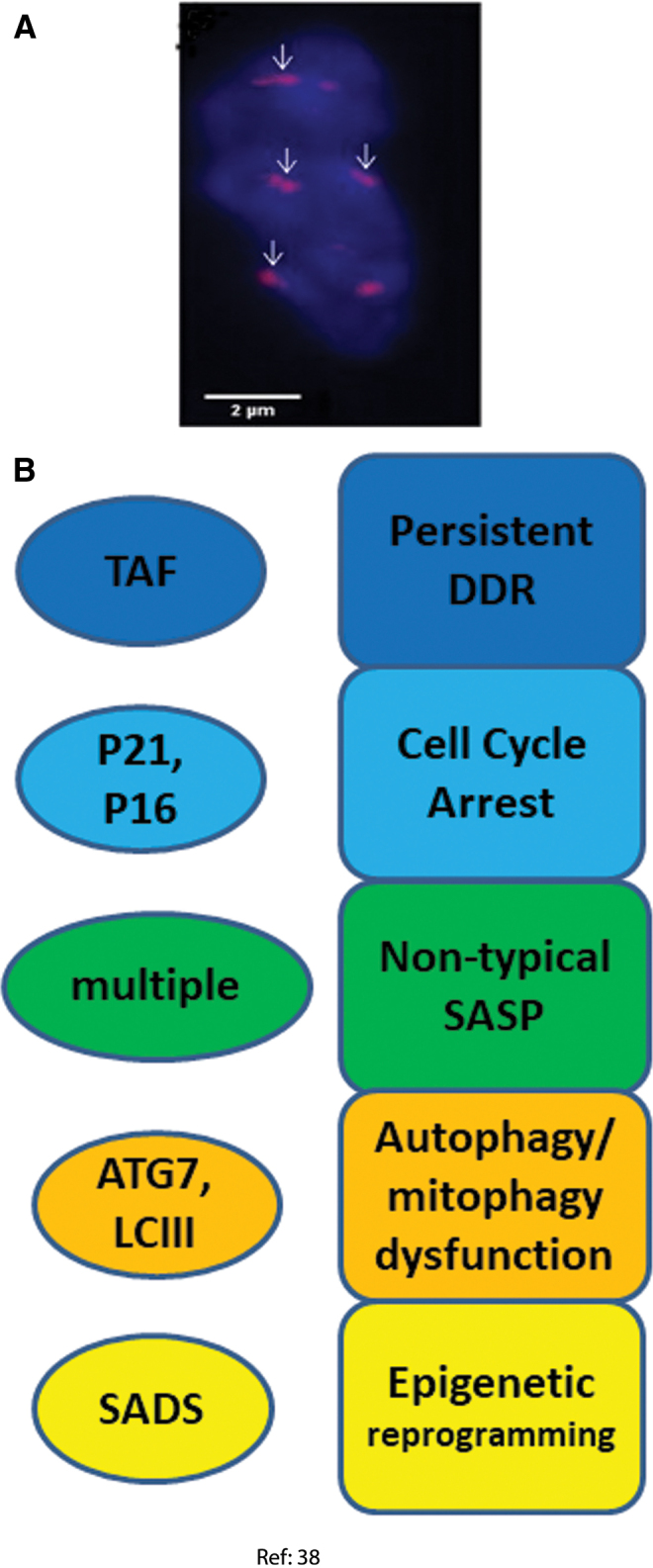
**Senescence markers measured in osteocytes. (A)** SADS (*arrows*) in an osteocyte freshly isolated from bone marrow of old (24 months) mice (38). **(B)** Senescence markers observed and senescence phenotypes inferred in osteocytes (38). Color images are available online.

**FIG. 6.**
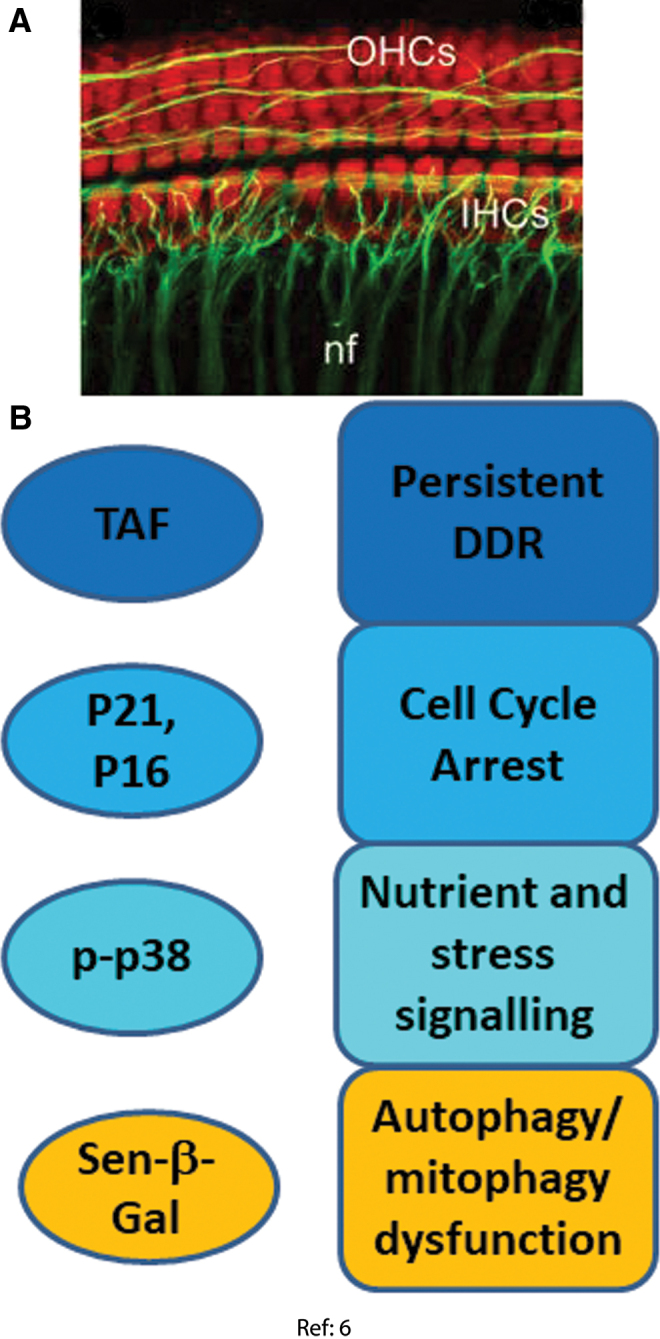
**Senescence markers measured in post-mitotic cochlear cells. (A)** Basal region of cochlear explant stained with myosin 7A (*red*, outer hair cell [OHC] and inner hair cell [IHC]) and with neurofilament 200 (*green*, auditory nerve fibers, nf) (6). **(B)** Senescence markers observed and senescence phenotypes inferred in cochlear cells (6). Color images are available online.

Here, we focus on the question as to what extent a full senescent phenotype has been confirmed in post-mitotic cell types by assessing markers covering the majority or all of the senescence domains indicated earlier. Studies that showed only upregulation of senescence markers in whole tissues without discriminating between post-mitotic and mitotically competent cells were considered only in exceptional cases as confirmatory evidence. We also excluded *in vitro* studies, as these were generally performed in differentiating stem cell systems, where a clear distinction of proliferation-competent progenitor cells and post-mitotic, terminally differentiated cells is often far from trivial. Results are summarized in Figures 2–6.

### Neurons

Our group previously analyzed the abundance of multiple senescence markers in cerebellar Purkinje neurons, cortical neurons, and neurons of the myenteric plexus during aging of mice between 4 and 32 months (60). More recently, brain neuron senescence was also observed in neurons bearing tau neurofibrillary tangles (NFT) from Alzheimer's disease patients as well as in the brains of an Alzheimer's disease mouse model with elevated tau expression (98). Moreover, senescence phenotypes were reported in the mouse retinal ganglial cell layer under ischemia (104). Colocalization of senescence signals with neuronal markers was shown in two of the studies (60, 104). In addition, Musi *et al.* (98) used microdissection of NFT-bearing human neurons to show transcriptional upregulation of TGFβ-, p38MAPK-, NF-κB-, and p53-regulated genes, and they showed consistent tracking of multiple senescence markers with NFT burden in human disease and in multiple mouse models, including mice treated with senolytics (98). Jurk *et al.* showed co-localization of multiple senescence markers in the same neurons (60). Interestingly, in the oxygen-induced retinopathy model, retinal ganglion neurons were the first cell type to present senescence markers after stress, and senescence was spread from neurons to retinal microglial cells and the vasculature by bystander signals (104). The following senescence domains were assessed in these studies (Fig. 2).

#### DDR and cell cycle arrest markers

All three studies used γH2A.X as a DDR marker, which was shown to co-localize with neuronal markers by Jurk *et al.* and Oubaha *et al.* (60, 104), whereas p53 target genes were elevated in NFT-bearing neurons (98). γH2A.X-positive Purkinje neurons were shown to be also positive for the lipid peroxidation product 4-hydroxynonenal (4-HNE), suggesting co-activation of DDR and SAMD (60), whereas γH2A.X co-localized with promyelocytic leukemia protein bodies in retinal ganglion cells (104). A strong γH2A.X signal in Purkinje and cortical neurons was driven by dysfunctional telomeres and was dependent on intact p21 (60). γH2A.X levels and CDKN2A (p16) expression correlated with NFT burden in both human and tau-transgenic mouse brains (98). In retinal ganglion neurons, CDKN1A and CDKN2A expression and p53, p16, and γH2A.X protein abundance were elevated after ischemia (104).

#### Senescence-associated secretory phenotype

The proportion of Purkinje and cortical neurons with a positive staining signal of proinflammatory cytokine interleukin (IL)-6, a classic SASP member, was found to increase with age in mouse brain neurons and IL-6-producing neurons were shown to be also positive for the oxidative damage marker 4-HNE (60). NF-κB, IL-β, and CXCL1 were transcriptionally upregulated in NFT-bearing human neurons (98). Oubaha *et al.* (104) found increased expression of multiple SASP marker genes in the retinal ganglion cells, including matrix-degrading enzyme plasminogen activator inhibitor 1 (Pai1), TGF-β1, IL-6, IL-1β, and vascular endothelial growth factor α. Retinal ganglion cell SASP was dependent on semaphorin 3A (SEMA3A) expression, and suppression of SEMA3A was able to block paracrine propagation of senescence (104).

#### Senescence-associated mitochondrial dysfunction

In old mice, Purkinje and cortical neurons accumulated the lipid peroxidation product 4-HNE and more ROS were produced in enteric neurons as shown by increased fluorescence from the ROS indicator dye dihydrorhodamine-123 (60). Moreover, increased autofluorescence in brain neurons of old mice indicated accumulation of lipofuscin, another indicator of oxidative stress and damage (13, 14). In hippocampus and cortex of NFT-bearing tau transgenic mice, complex I- and -II-linked ATP production was decreased without change of mitochondria mass (98), indicating impaired mitochondrial function. Although the study by Oubaha *et al.* (104) used oxidative stress to induce retinopathy and senescence, SAMD indicators were not measured.

#### Autophagy dysfunction

Senescence-associated beta-galactosidase (SA-β-Gal) reporting lysosomal overload and thus the possibility of autophagy dysfunction, is a frequently used marker for senescence and was assessed in all three studies. SA-β-Gal staining was enhanced in brain, mesenteric and retinal ganglion neurons (60, 104). Although no SA-β-Gal staining was found in NFT-bearing mouse brains, the expression of the GLB1 gene encoding for β-galactosidase was enhanced (98). In addition, the previously mentioned lipofuscin accumulation (60) can also be considered as a marker for autophagy dysfunction as lipofuscin accumulation inhibits both autophagic flux and proteasomal protein degradation (53, 122, 124).

#### Nutrient and stress signaling

p38MAPK is a sensor and responds to a wide range of different stresses, which can initiate senescence (41). High levels of phosphorylated p38MAPK were observed in neurons in aging mice, especially those that were also positive for 4-HNE (60), and p38MAPK and downstream regulated genes were upregulated in NFT-bearing human neurons (98). In retinal ganglion neurons, p38MAPK was not assessed but high levels of phosphorylated inositol-requiring enzyme 1α (IRE1α), a marker for endoplasmic reticulum (ER) stress, were observed (104). In fact, retinal senescence was dependent on the endoribonuclease activity of IRE1α (104). ER stress had been implicated in different senescence modes, including oncogene HRAS^G12V^-induced senescence (35) and of chondrocyte senescence in osteoarthritis (77). Markers of nutrient signaling have to our knowledge not been assessed in the context of neuron senescence.

#### Epigenetic modification

Markers for epigenetic reprogramming in neurons were only assessed in one of the studies. Jurk *et al.* (60) showed enhanced nuclear granularity for the heterochromatin-associated histone variant mH2A in Purkinje and cortical neurons from old mice.

### Cardiomyocytes

Recent studies indicate that human and mouse cardiomyocytes retain the ability to divide, but do so very rarely leading to turnover rates of <1% in adulthood (7, 111, 118). A recent paper showed that mouse and human cardiomyocytes acquire senescence-like phenotypes during aging from 3 to 30 months in mice and from adult (46–65 years) to old (74–82 years) age in humans. This was characterized by persistent DNA damage at telomeres, p21 and p16 activation, and a noncanonical SASP (4). In detail, the following senescence domains were considered (Fig. 3).

#### DDR and cell cycle arrest markers

Telomere-associated DNA damage foci (telomere-associated foci [TAF]) can be instigated by DNA damage even at long telomeres and are a persistent form of DNA damage (42, 51) and thus potent inducers of a senescent phenotype (61). They were found to trigger senescence regardless of telomere length both *in vivo* in old mouse and human hearts and *in vitro* in cultivated mouse embryonic cardiomyocytes. Independence of telomere length strongly indicated that the cardiomyocytes that underwent this type of aging were, in fact, post-mitotic (4). Increased TAF frequencies were paralleled by enhanced transcription of cell cycle arrest markers p21, p16^INK4a^, and p15^INK4b^ and increased p21 protein abundance in cardiomyocytes from old mice. Transgenetically induced telomeric DNA double-strand breaks remained persistent and were able to induce further markers of senescence in cardiomyocytes, whereas non-telomeric DNA damage was repaired within a few hours and did not cause a senescent phenotype (4).

#### Autophagy dysfunction and cellular morphology change

Increased SA-β-Gal activity was found in the hearts of old mice and was localized solely to cardiomyocytes (4), which showed that autophagy dysfunction occurred specifically in the post-mitotic cells. Telomeric DNA double-strand breaks, induced in cultured rat neonatal cardiomyocytes after transfection with a telomere-targeting endonuclease, were sufficient to induce increased SA-β-Gal activity (4). Moreover, cardiomyocyte hypertrophy was found *in vivo* in old mice and *in vitro* in cultivated rat neonatal cardiomyocytes under all senescence-inducing conditions, possibly due to elevated Myh7 and Acta1 gene expression (4).

#### Senescence-associated mitochondrial dysfunction

Mitochondrial dysfunction was evidenced by decreased expression of many mitochondrial genes, especially those related to mitochondrial inner membrane and electron transport chain (4). Expression of MnSOD (SOD2) and catalase was reduced in old cardiomyocytes, whereas the expression of monoamine oxidase A, an enzyme promoting oxidative stress, was increased (4). Further, frequencies of cardiomyocytes positive for the markers of ROS-mediated damage 4-HNE and 8-oxodG were also increased in old mice (4).

#### Senescence-associated secretory phenotype

Classical proinflammatory SASP markers such as IL-6 and CXCL1 were found to be elevated in old mice whole hearts (103) but not in purified cardiomyocytes showing telomeric DNA damage, SA-β-Gal activity, and SAMD. In contrast, these cells displayed a noncanonical SASP characterized by enhanced expression of Edn3, TGFβ2, and Gdf15 with enhanced Edn3 expression specific to old cardiomyocytes. This SASP (especially TGFβ2) was antiproliferative, profibrotic, and prohypertrophic in co-cultured cells (4).

#### Epigenetic modification

Senescence-associated distension of satellites (SADS) has been suggested to be an early chromatin modification in senescence, which frequently occurs in replicative and oncogene-induced senescence (25). Increased frequencies of SADS-positive cardiomyocytes were found in the hearts of old mice (4).

### Skeletal muscle myofibers

In the context of a study of the bystander effects of senescent cells *in vivo*, our group recently reported the induction of multiple markers of senescence in gastrocnemius and biceps femoris muscle of mice aging from 8 to 32 months. Myofiber senescence marker correlated with low fiber diameter, an indicator of muscle aging and sarcopenia at both interindividual and single fiber levels (28). A limitation of this study is that myofiber nuclei were only identified by their position within a fiber as outlined by wheat germ agglutinin staining, but satellite cells were not excluded by positive staining with a stem cell marker. However, satellite cell frequencies were regarded as too low to significantly falsify counts of myofiber nuclei bearing senescence markers. The following senescence domains were assessed (Fig. 4).

#### DDR and cell cycle arrest markers

In old mice skeletal muscles, there was a higher frequency of TAF-positive nuclei as well as a tendency toward more p21-positive nuclei (28). This result was strengthened by an experiment, in which senescent cells were xenotransplanted into muscle of immunodeficient NOD scid gamma mice. In this experiment, significant higher frequencies of TAF- or p21-positive myofiber nuclei were found in the vicinity of the transplanted senescent cells, but not next to nonsenescent transplanted cells. Moreover, p16 and p21 mRNAs were increased in old muscles.

#### Senescence-associated secretory phenotype

The proinflammatory SASP genes IL-1α, IL-1β, IL-6 and tumor necrosis factor alpha (TNF-α) were enhanced at mRNA level in old muscles. However, no evidence was found for enhanced protein levels of proinflammatory cytokines in either muscles from old mice or in the myofibers in muscles transplanted with senescent cells. Reasons for this discrepancy were not clear, and potential non-proinflammatory SASP components were not evaluated in this study (28).

#### Senescence-associated mitochondrial dysfunction

The article used Sudan Black B (SBB) staining as an indicator of oxidative damage as described (44). However, in skeletal muscle, the SBB staining pattern is primarily determined by fiber type, with oxidative fibers positive for SBB in contrast to glycolytic fibers. Therefore, a reliable assessment of mitochondrial dysfunction as stress response in any fiber type has not yet been performed.

#### Epigenetic modification

Nuclear exclusion of HMGB1 and decrease of LB1 at the nuclear lamina are well-established senescence markers (32, 40). Both decreased HMGB1 nuclear staining and increased heterogeneity of the LB1 distribution over the nuclear lamina were found in myocyte nuclei of old mice. LB1 heterogeneity over the nuclear lamina was also increased in the vicinity of xenotransplanted senescent cells (28).

### Osteocytes and osteoblasts

Farr *et al.* separately isolated T cells, myeloid cells, osteoblast progenitors, and, important in the present context, mature post-mitotic osteocytes and osteoblasts from bone marrow in young (6 months) and old (24 months) mice (38). A range of senescence markers was measured in these fractions without any *in vitro* subculturing of the cells (Fig. 5). In general, increases of senescence markers observed in post-mitotic osteoblasts and osteocytes were at least comparable in magnitude if not larger than those seen in progenitor and hematopoietic lineage cells.

#### DDR and cell cycle arrest markers

The percentage of TAF-positive osteocytes increased from <20% in young mice to almost 90% in old ones together with an increase in p21 gene expression. p16^Ink4a^ mRNA levels were found to increase dramatically in both osteocytes and osteoblasts isolated from trabecular and cortical bones of both old female and male mice. In addition, p16^Ink4a^ and p21 mRNA expression was also increased in bone biopsies of old female human donors, although this analysis did not distinguish cell types (38).

The expression of a panel of 36 established SASP genes was investigated by Real Time quantitative polymerase chain reaction (RT-qPCR) on different types of cells isolated from old and young mouse bones (1, 22, 23). Very few of these SASP factors were significantly altered in B cells, T cells, osteoblast progenitors, or osteoblasts. However, 23 of the 36 SASP genes analyzed were significantly increased in old *versus* young osteocytes. Twelve SASP genes out of the same 36 gene panel were also enhanced (at *p* < 0.05) at whole tissue level in bone biopsies from old *versus* young human donors, although an enrichment analysis for the whole gene set did not result in statistical significance (38).

#### Autophagy dysfunction

The expression of two autophagy marker genes, Atg7 and Map1lc3a (commonly known as LC3) was measured by RT-qPCR. Lower expression of both in osteocytes (but not osteoblasts) from old mice was found (38).

#### Epigenetic modification

The presence of SADS was analyzed as a marker for chromatin reorganization in senescence, and an increase from 2% to 11% of SADS-positive osteocytes was found in the bone cortices of old mice (38).

### Cochlear hair cells

Recently, Benkafadar *et al.* reported the upregulation of multiple markers of cell senescence in the cochlea of prematurely aging SAMP8 mice (6). Proliferation-competent support and progenitor cells are present in the cochlea, whereas major cell types are the post-mitotic sensory hair cells and ganglion neurons. Some of the analyzed markers (*e.g.*, SA-β-Gal staining) were localized by the authors to the hair cells in the organ of Corti and to spiral ganglion neurons, whereas others were only assessed in whole tissue homogenates. However, the authors also studied oxidative stress-induced senescence in explanted and *in vitro* cultured cochleae and organs of Corti, again showing preferential senescence induction in hair cells, especially those of the outer layer. Markers for the following senescence domains were assessed (Fig. 6).

#### DDR and cell cycle arrest markers

Significant increases of p21, p16, p53, and p-Chk2 protein levels were found in whole cochlear extracts from SAMP8 mice compared with slow-aging SAMR1 mice starting from 6 to 12 months of age (6). Activation of a DDR in the hair cells was confirmed in the *ex vivo* model, showing increased numbers of γH2A.X and 53BP1 foci in outer and inner hair cell nuclei at 3 days after oxidative stress. This result was confirmed by Western blot on whole cochlear extracts (6). Moreover, higher levels of the DNA nucleotide excision repair protein DDB2 (DNA binding protein 2) were also found in these cells, associated with increased levels of phosphorylated Chk2, p53, and p21 (6).

#### Senescence-associated mitochondrial dysfunction

In whole cochlear extracts from aged SAMP8 mice, lower protein levels of MnSOD and the oxidant resistance regulator Nrf2 and higher levels of p66Shc and phospho-p66Shc, a negative regulator of MnSOD (69) whose upregulation can lead to higher oxidative stress (46), were found. In isolated cochlear extracts at 3 days after oxidative challenge, MnSOD and catalase protein and activity were enhanced, as were p66Shc and phospho-p66Shc and malondialdehyde content, indicating persistently increased oxidative stress. Whether this was associated with mitochondrial dysfunction as in fibroblast stress-induced senescence (106) has not been established.

#### Autophagy dysfunction

SA-β-Gal activity was associated with inner and outer hair cells and spiral ganglion cells in both *in vitro* cultured cochlea after oxidative stress and in cochleae from aged SAMP8 mice. In addition, higher levels of phosphorylated Beclin 1, Rab7, and LC3 II were found in cochlear extracts of old SAMP8 mice and in stressed cochlea *in vitro* (6). These results suggested an increased autophagy with inefficient lysosomal flux clearance, indicating autophagy dysfunction.

#### Stress signaling

At 3 days after oxidative stress, cochlear hair cells became senescent, marked by the increased levels of p38 and its phosphorylated form (6).

### Conclusions

Three independent laboratories have shown different types of neurons expressing markers for multiple senescence domains under different age-related stressors. Markers for each of the senescence-associated domains shown in Figure 1 were found in stressed and/or aged neurons (Fig. 2), and some of these markers were co-localized in the same cells (60). Altogether, the data provide robust evidence that neurons do respond to age- or disease-associated stress by senescence, even when they have long before ceased to proliferate.

Evidence related to cell senescence in other post-mitotic cell types is less strong. Results per cell type have not yet been independently confirmed. Markers for four to six of the seven senescence domains identified in Figure 1 were assessed in each of the examined non-neuronal post-mitotic cell types (Figs. 3–6). Only DDR markers were measured in all cell types and were found to be consistently activated. Secretions of the canonical proinflammatory SASP were not confirmed in heart nor skeletal muscles, and there could be cell-type specific differences. On the other hand, variations of the SASP have been observed as well during senescence of proliferation-competent cells, indicating that the SASP is modified by a multiplicity of factors, including engagement of check-point proteins p53, p21, and p16, mitochondrial (dys) function, response kinetics, and cell type (17, 52). Together, we feel that the evidence is good enough to conclude that probably all post-mitotic cells have the ability to mount a senescence response, and that they probably do so in measurable quantities during aging and under physiologically and pathologically relevant stresses.

## How Important Is Senescence of Post-Mitotic Cells to Understand Mammalian Ageing *In Vivo*?

The pathogenic roles of senescent cells are now well recognized. Growing evidence shows that selective elimination of senescent cells, or reducing the SASP and thus senescence-induced bystander effects, improves a wide range of age-associated and/or pathologic conditions (121). This may also hold for post-mitotic cell senescence. For example, in a model of ischemic retinopathy, retinal ganglion cells became senescent and the resulting SASP caused pathologic angiogenesis, which worsened the retinopathy. Metformin, which reduced the SASP, prevented the adverse effects of ischemic retinopathy (104). It is less clear whether and to what extent post-mitotic senescent cells affect normal tissue physiological functions, especially aging. However, there are striking similarities between cell senescence, including post-mitotic senescence, and aging phenotypes as observed in tissues composed to a large extent of post-mitotic cells. It might thus be speculated that a better understanding of the cellular regulatory pathways that govern the senescent stress response might lead to a deeper comprehension of, and better possibilities for, interventions into age-associated functional decline in post-mitotic tissues. Keeping in mind the present limitations of knowledge, we will now discuss some of the possible relevance of post-mitotic senescence for aging, using myofiber senescence and muscle aging as an example.

### Ageing of skeletal muscle

Skeletal muscle not only has a primary role in locomotion and in the maintenance of posture but also shapes metabolic homeostasis by taking up glucose and oxidizing fatty acids. There are four major fiber types in mammalian muscle: slow (type 1) and three fast types (2A, 2X, and 2B). Each type is characterized by the expression of one specific isoform of the myosin heavy chain, which is the main determinant of their contractile properties. Fiber types differ in their metabolic profiles, ranging from slow/oxidative to fast/glycolytic (116).

One prominent change in skeletal muscle during aging is the decline of skeletal muscle mass and function. It leads to gait instability and increased risk of falls (137) and is a primary cause of sarcopenia, a leading cause of death in elderly (68). By age 70, the mean cross-sectional area of skeletal muscle is reduced by 25%–30% and muscle strength diminishes by 30%–40% (109). These changes are believed to be both due to a decrease in the number of muscle fibers and due to atrophy and weakening of those remaining (12, 73, 74).

Age-related sarcopenia in humans affects fast fibers more strongly than slow ones (3, 72). Maintenance of muscle mass is achieved by anabolic and catabolic balance. The main anabolic stimulation comes from muscle contraction to which the muscle cells respond by adaptive mechanisms. Resistance exercise is known to counteract the loss of muscle mass and function; however, aged muscle fails to readily adapt to exercise and this anabolic resistance is proposed to play a major role in sarcopenia.

Mitochondrial activity in skeletal muscles has long been known to correlate with exercise capacity (39), with a recent observation showing that especially with respect to complex I (43), suggesting mitochondrial function is critical for skeletal muscle function. Mitochondrial content has widely been observed to decrease with age in skeletal muscles (58, 78, 97). Skeletal muscle mitochondria also undergo functional changes with age; respiratory coupling decreases, and the rate of ROS release per unit of mitochondrial protein increases (49, 83). Therefore, decreases in both quality and quantity of mitochondria contribute to the overall loss of mitochondrial function in aged skeletal muscles.

### Is post-mitotic senescence relevant for skeletal muscle aging?

Senolytic treatment was shown to be effective in preserving muscle fiber diameter in skeletal muscles (5). Mechanistically, this has been assumed to be caused by either systemic reduction of SASP factors or improved stem (satellite) cell function. However, do senescent myocytes also account for development of sarcopenia and other features of aged skeletal muscles?

Our current understanding of the nature of post-mitotic senescence in general and in muscle specifically is limited. This poses a number of unanswered questions. For instance, what are the actual proportions of cells that senesce in different muscles? Are different myofiber types differently susceptible to senescence? There appears to be a cellular state of transition from normal to senescent (106), but how long does this transition last in myofibers *in vivo*? How efficient is immunosurveillance of senescence in muscle? How long do senescent myofibers persist in the tissue? When senescent cells were implanted into skeletal muscles in immune-deficient mice, neighboring cells became senescent within about 1 month (28). However, during normal, healthy aging *in vivo* endogenous stress factors might be different and the immune system is generally competent, leading to possibly a different time course of senescence development.

Despite these limitations, it appears well possible that senescent myocytes may account for the development of sarcopenia in multiple ways. Importantly, an association between the presence of senescence markers in myocyte nuclei and low fiber diameter in the vicinity has been established (28). During mammalian aging, fast glycolytic fibers undergo a significant size reduction in aging, unlike slow fibers (97). Interestingly, our preliminary data in a premature aging mice model show higher frequencies of nuclei bearing the senescence marker telomere-associated DNA damage foci in glycolytic fibers than in slow/oxidative fibers. However, it is unknown whether induction of a senescent stress response caused fiber atrophy or conversely, was due to it.

The main anabolic stimulation important for maintenance of muscle mass derives from muscle contraction to which the muscle cells respond. These adaptive response mechanisms include those through redox-signaling pathways medicated by contraction-mediated increases in the generation of superoxide and nitric oxide by skeletal muscle fibers (85). However, aged muscle fails to adapt to exercise mainly because of an attenuated response to ROS-stimulated redox signaling, and this anabolic resistance is proposed to play a major role in sarcopenia. Elevated levels of oxidative stress cause the chronic activation of multiple signaling pathways, contributing to the blunted response of aged muscle to contraction-medicated signals. In both single fibers and isolated mitochondria from skeletal muscles, ROS were found to increase with age (83, 105). Although there are no empirical data yet on ROS levels specifically in senescent myocytes, elevated ROS production in senescent cells, in general, is well documented (61, 93, 106). Thus, it is very possible that senescent myocytes contribute to the elevated oxidative stress in aging muscle tissue. However, as we do not know the proportion of senescent myocytes in aged skeletal muscles, we cannot yet establish the relative importance of the contribution of senescent myocytes to oxidative stress in aged muscles.

Mitochondrial dysfunction (SAMD), characterized by increased mitochondrial biogenesis, increased levels of ROS, and reduced coupling (106) is a major senescence domain. It is related to decreased mitophagy (30, 64). In isolated mitochondria from skeletal muscles of aged animals, functional properties consistent with a typical SAMD were observed, namely increased ROS release and decreased coupling (49, 83). Dietary restriction and dietary restriction mimetics, including rapamycin, reduced mitochondrial ROS levels and improved mitochondrial coupling (92, 93). Correspondingly *in vitro*, rapamycin reduced both SAMD and SASP in senescent fibroblasts (24, 34).

In fibroblast cell senescence *in vitro*, increased levels of mitochondrial biogenesis markers such as PGC-1α/β and increased mitochondrial mass were observed (70, 106). However, in contrast, in aged skeletal muscles, decreased mitochondrial mass in both fast and slow fibers was found (97). Currently, it is unclear whether mitochondrial mass increase is a universal feature of the senescent phenotype. A decrease in mitophagy seems to be the essential factor causing mitochondrial mass increase in senescing fibroblasts (30). An experimental assessment of the balance between mitochondrial biogenesis and mitophagy during myofiber senescence will be necessary to clarify this point.

SASP is another senescence domain that might also contribute to sarcopenia. In fact, inflammation has been proposed as a key driver of skeletal muscle aging (29, 85). For example, exposure of skeletal muscle to TNF-α results in muscle weakness associated with a loss of total muscle protein through increased NF-κB activation, at least partly mediated by ROS signaling (76). In aged human muscle, increased levels of NF-κB have been suggested as a contributing cause to the anabolic resistance in aging (113). However, the supporting data are inconsistent, because some papers reported increases in gene expression levels for proinflammatory cytokines in human skeletal muscles with age (86, 88, 110), whereas others did not (89). We found elevated gene expression levels of proinflammatory cytokines IL-1α, IL-1β, IL-6, and TNF-α in old mice skeletal muscles but no differences to young animals at protein level (28). In cardiomyocytes, an atypical antiproliferative, non-inflammatory SASP was found (4). The composition of the SASP in senescent skeletal myofibers, and its potential contribution to chronic inflammation in aged muscle still needs to be established. Moreover, skeletal muscle has recently been proposed to be a potential source of a diverse range of cytokines, termed myokines (102, 134). Exercise-stimulated myokines, such as IL-15, can have beneficial effects on the immune system and on control of adiposity (102). Whether myofiber senescence impacts myokine expression and secretion, and the role of myokines in skeletal muscle aging are important topics for future studies.

## Conclusions

There is good evidence for a senescent phenotype as stress response in post-mitotic cells, including skeletal myofibers. It is possible that post-mitotic cell senescence has functional relevance in tissue aging, but the evidence for this is still weak. Important open questions are:

1. The phenotype of senescent myofibers needs to be better characterized. How do they differ from fibroblasts? What are the functional implications? Surprisingly little evidence exists for senescent myofibers or old muscle tissues being proinflammatory.
2. The actual proportions of senescent myocytes in aging muscles need to be determined. This requires improved quantitative techniques for senescent cell detection *ex vivo*.
3. Systemic senolytic intervention has been shown to be effective in postponing sarcopenia (5, 140). As SAMD is an important senescence domain, this might have resulted in improved mitochondrial function *in vivo*. Senolytic or senostatic interventions might have better potential to improve mitochondrial function than many other drugs that increase mitochondrial biogenesis. The impact of anti-senescence interventions on muscle mitochondrial function needs to be assessed.

## Abbreviations Used

4-HNE: 4-Hydroxynonenal
ATM: Ataxia telangiectasia mutated
ATR: ATM and RAD3-related
CDK: cyclin-dependent kinase
CI: complex I of the mitochondrial electron transport chain
DDF: DNA damage foci
DDR: DNA damage response
ER: endoplasmic reticulum
IL: interleukin
IRE1α: inositol-requiring enzyme 1α
LB1: lamin B1
mTOR: mammalian target of rapamycin
mTORC: mTOR complexes
NFT: neurofibrillary tangles
NF-κB: nuclear factor-κB
PML: Promyelocytic leukemia protein
ROS: reactive oxygen species
RS: replicative senescence
RT-qPCR: Real Time quantitative polymerase chain reaction
SA-β-Gal: Senescence-Associated beta-Galactosidase
SADS: senescence-associated distension of satellites
SAHF: senescence-associated heterochromatin foci
SAMD: senescence-associated mitochondrial dysfunction
SASP: senescence-associated secretory phenotype
SBB: Sudan Black B
SCARS: DNA segments with chromatin alterations reinforcing senescence
SEMA3A: semaphorin 3A
SIPS: stress-induced premature senescence
TAF: telomere-associated foci
TGFβ: transforming growth factor beta
TL: telomere length.
TNF-α: tumor necrosis factor alpha
